# Ultrafast electron-phonon coupling and photo-induced strain in the morphotropic phase boundary of Bi_x_Dy_1−x_FeO_3_ films

**DOI:** 10.1038/s41598-018-21655-9

**Published:** 2018-02-19

**Authors:** Zeyu Zhang, Lu You, Juan Du, Junling Wang, Zuanming Jin, Guohong Ma, Yuxin Leng

**Affiliations:** 1 State Key Laboratory of High Field Laser Physics, Shanghai Institute of Optics and Fine Mechanics, Chinese Academy of Sciences, Shanghai, 201800 China; 2 Department of Physics, Shanghai University, Shanghai, 200444 China; 3 School of Materials Science and Engineering, Nanyang Technological University, Singapore, 639798 Singapore

## Abstract

The interplay among ferroelectric, magnetic and elastic degrees of freedom in multiferroics is the key issues in condensed matters, which has been widely investigated by various methods. Here, using ultrafast two-color pump-probe spectroscopy, the picosecond electron-phonon and spin-lattice coupling process in Dysprosium doped-BiFeO_3_ (BDFO) films on SrTiO_3_ (STO) substrate have been investigated systematically. The Dy-doping induced structural transition and magnetic enhancement in BDFO is observed by ultrafast electron-phonon and spin-lattice interaction, respectively. The elastic anomalies in BDFO films are revealed by the photo-induced coherent acoustic phonon. With increasing the Dy doping ratio, the frequencies of the acoustic phonon in the films are modulated, and the phonon transmission coefficient between films and substrate is found to approach unity gradually. The ultrafast observation of the tunability of the ferroelectric, magnetic and the elastic properties in the morphotropic phase boundary of rare-earth doped BFO films provides new insights into the integration of BFO in next-generation high frequency electro-magnetic and electroacoustic devices.

## Introduction

As a monophase multiferroic, bismuth ferrite (BFO) shows coexistence of both antiferromagnetic and ferroelectric behavior at room temperature, which had been widely investigated in the past decade^1–3^. The bulk BFO crystal has a rhombohedral structure at room temperature. For a compressively strained BFO film, it could be transformed to be a supertetragonal phase, which could result in strongly enhanced ferroelectricity and ferromagnetism^4–6^. From application point of view, partial substitution of Bi with a rare earth element such as Dy in BFO system was reported to stabilize the ferroelectric perovskite phase, which can bring a reduction in the leakage current and enhancement in the net magnetic moments for BFO^7–11^. In addition, BFO exhibits some exotic photo-induced effects such as photovoltaic effect^12^, ultrafast photoinduced strain^13^, ultrafast THz radiation^14^, giant electrochromic behavior and photostriction properties^6,15,16^. Recently, the ultrafast electron-magnon coupling and giant ultrafast photo-induced strain were reported in BFO^17^, which draws new insight into the ultrafast electric-magnetic-elastic coupling in this type of material.

Ultrafast spectroscopy has been employed to investigate the complicated phase transition and coupling among electron, lattice and spin subsystem in multiferroics, which shows possible magnetoelectric coupling occurring at the vicinity of the transition temperature^18^. Meanwhile, the electromechanical coupling in multiferroics also has been paid a special attention for the potential applications in piezoelectric or electrostrictive actuators, sensors and energy harvesting devices, etc^19,20^. It’s reported that by means of dysprosium substitution, the crystal structure of BFO films could be modulated from rhombohedral to orthorhombic, which could result in the releases of the compressive strain introduced by the mismatch between the BFO film and SrTiO_3_ (STO) substrate^21^. Here, ultrafast pump-probe spectroscopy is employed to investigate a series of Bi_x_D_1−x_FeO_3_ (BDFO) thin films, the ultrafast dynamics of electron-lattice and spin-lattice response is found to be sensitive to the phase transition around the critical point, a sub-picosecond electric fields applied to films for generating ultrafast coherent acoustic phonons, which shows the ultrafast piezoresponse softening behavior at the phase boundary. These results shed a new light on the chemical and strain engineering of the ultrafast electromechanical coupling in BFO films.

## Results and Discussion

**Figure 1:**
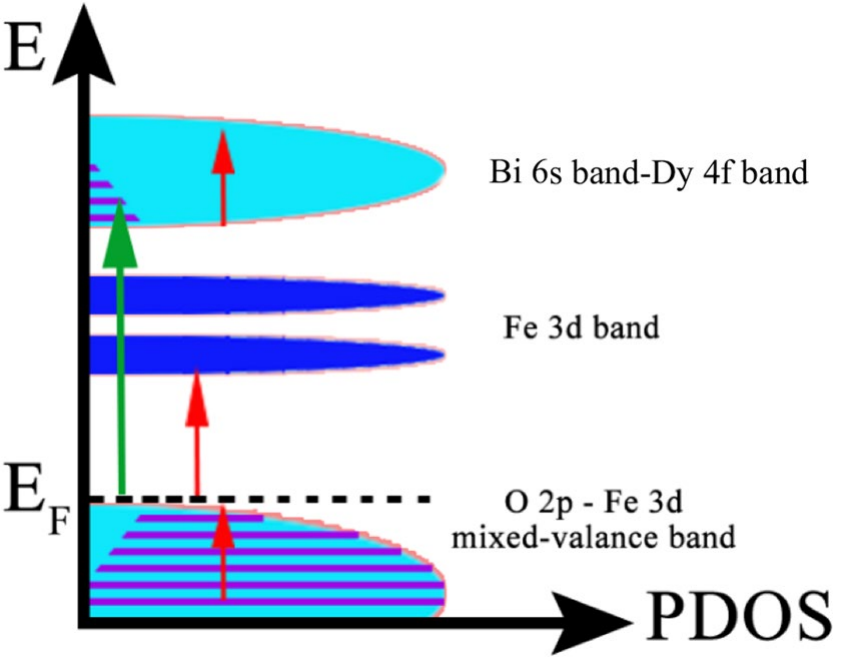
The diagram of optical transition in BDFO pumped by 3.1 eV femtosecond pulse.

**Figure 2:**
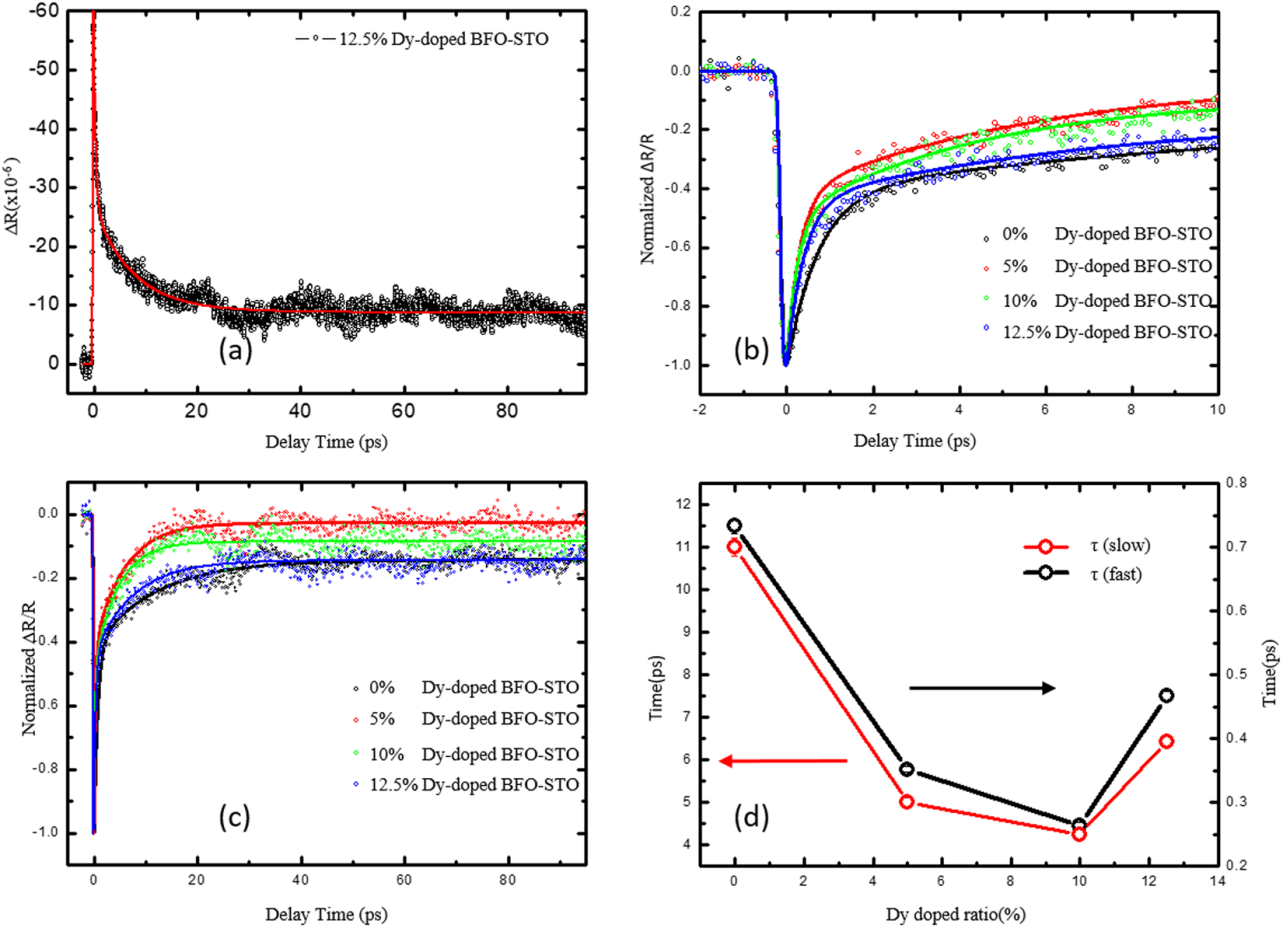
The typical pump-probe reflection spectroscopy with pumping fluence of 4 mJ/cm^2^, the solid red line is bi-exponential fitting (**a**). Normalized transient reflectivity of BDFO thin films with different Dy doping ratio in time scale of 10 ps (**b**), and in 100 ps (**c**). The Dy-doping ratio dependence of fitting time constants for spin-lattice coupling (red dot) and electron-lattice coupling (black dot), respectively (**d**).

It is known in plenty of the previous static spectroscopy works on BFO that the photoenergy of 3.1 eV pump beam is larger than the band gap of BDFO thin films^13,22,23^. As shown schematically in Fig. 1, when the 3.1 eV pump beam is irradiated on the surface of the sample, the hot electrons are pumped into the upper conduction band (formed by Bi^3+^ 5p-Dy^3+^ 4 f) from O^2−^ 2p- Fe^3+^ 3d mixed-valance band^18^. After the pump, as shown in Fig. 2(a), the transient reflectivity of the probe beam shows a sudden drop within 100 fs, and then relaxes back to a plat background longer than hundreds of picoseconds. The typical hot carrier relaxation process in BFO could be distinguished by two processes: 1) a fast component due to the electron-lattice coupling, and 2) a slow component contributed by the spin-lattice coupling. A convolution of the Gaussian pulse G(t) with a bi-exponential decay function is utilized to analyze the electron dynamics phenomenologically^13^,

1 $$\Delta R/R=({A}_{1}\exp (-\frac{t}{{\tau }_{e \mbox{-} ph}})+{A}_{2}\exp (-\frac{t}{{\tau }_{s \mbox{-} ph}})+{A}_{3}+{A}_{0}\,\cos (2\pi t/T+\varphi ))\otimes G(t)$$

where A_1_ and A_2_ are the amplitudes of the fast and slow components mentioned above, respectively. τ_e-ph_ and τ_s-ph_ are the relaxation time constants of the electron-phonon and spin-lattice interactions, respectively. A_3_ is the amplitude of the flat background, which recovers to the ground state after a much longer time delay (hundreds of picoseconds to several nanoseconds). A_0_ and T and ϕ are the amplitude, periodicity and initial phase of the acoustic phonons stimulated by the impulsive pumping pulse.

Figure 2(b,c) show ultrafast transient reflectivity of four BFO samples with four different Dy-substitution ratios at two different scanned time windows, respectively. In the first ten picoseconds shown in Fig. 2(b), the hot carriers collided and equilibrated with the lattice within ~0.5 ps, which depends on the concentration of Dy-substitution. It was reported that the electron-lattice coupling intensity is strongly related to the crystal structure^23^, therefore, the electron-lattice coupling change with Dy-doping concentration presents an evidence that the Dy-substitution could modify the crystal structure, and our experimental data is consistent with the reports in literatures^24^. After the first tens of picoseconds, the reflectivity relaxes to a plat background with a couple of shock waves, which arise from the photo-induced coherent acoustic phonon, as shown in Fig. 2(c). When the Dy-ratio increased from 5% to 12.5%, the magnitude of A_3_ is seen to increase gradually due to the suppression of the electric leakage properties. Figure 2(d) presents the extracted magnitudes of τ_fast_ and τ_slow_ for each Dy-ratio samples, As the Dy-ratio is increased from 0% to 10%, the electron-lattice coupling and spin-lattice coupling time is decreased gradually from 0.8 ps to 0.3 ps for τ_fast_, and 12 ps to 4 ps for τ_slow_, respectively. For the ultrafast interaction, the three-temperature model is employed to analyze the pump-probe response in BDFO films. After the initial pump process, the electron temperature is raised more than a thousand Kelvin, which renders the two sub-systems (electron and lattice) dropped into a nonequilibrium state^25^. The two sub-systems turn into equilibrium state through electron-lattice coupling within 1.0 picosecond, which is known as the electron-lattice coupling time (τ_fast_). Similarly, the lattice and the spin systems evolve into equilibrium in several picoseconds, which is the dynamical spin-lattice coupling process. When the structural phase transition of BDFO occurs, the magnetic state could be altered. As a result, the atomic lattice positions could be altered for the intrinsic spin-lattice interaction. The temperature dependence of the electron-lattice coupling time clearly shows the transition. From the TTM, the dynamical spin-lattice coupling timeτ_s-l_ is related to the lattice specific heat *C*_*l*_, spin specific heat *C*_*s*_, and spin-lattice coupling constant *G*_*sl*_ by

2 $${\tau }_{s \mbox{-} l}\approx \frac{{C}_{s}{C}_{l}}{{G}_{sl}({C}_{s}+{C}_{l})}.$$

The specific heat measurement of BDFO across the Néel temperature indicates that heat capacity of spin is much smaller than that of lattice, i. e. *C*_*s*_ << *C*_*l*_, therefore the expression above could be simplified to

3 $${\tau }_{s \mbox{-} l}\approx \frac{{C}_{s}}{{G}_{sl}}.$$

Thus, the enhancement of the dynamical spin-lattice coupling process represents the change of the spin state of BDFO in the structural phase transition temperature range independently, which is attributed to the magnetic enhancement by the Dy doping reported previously^8^.

**Figure 3:**
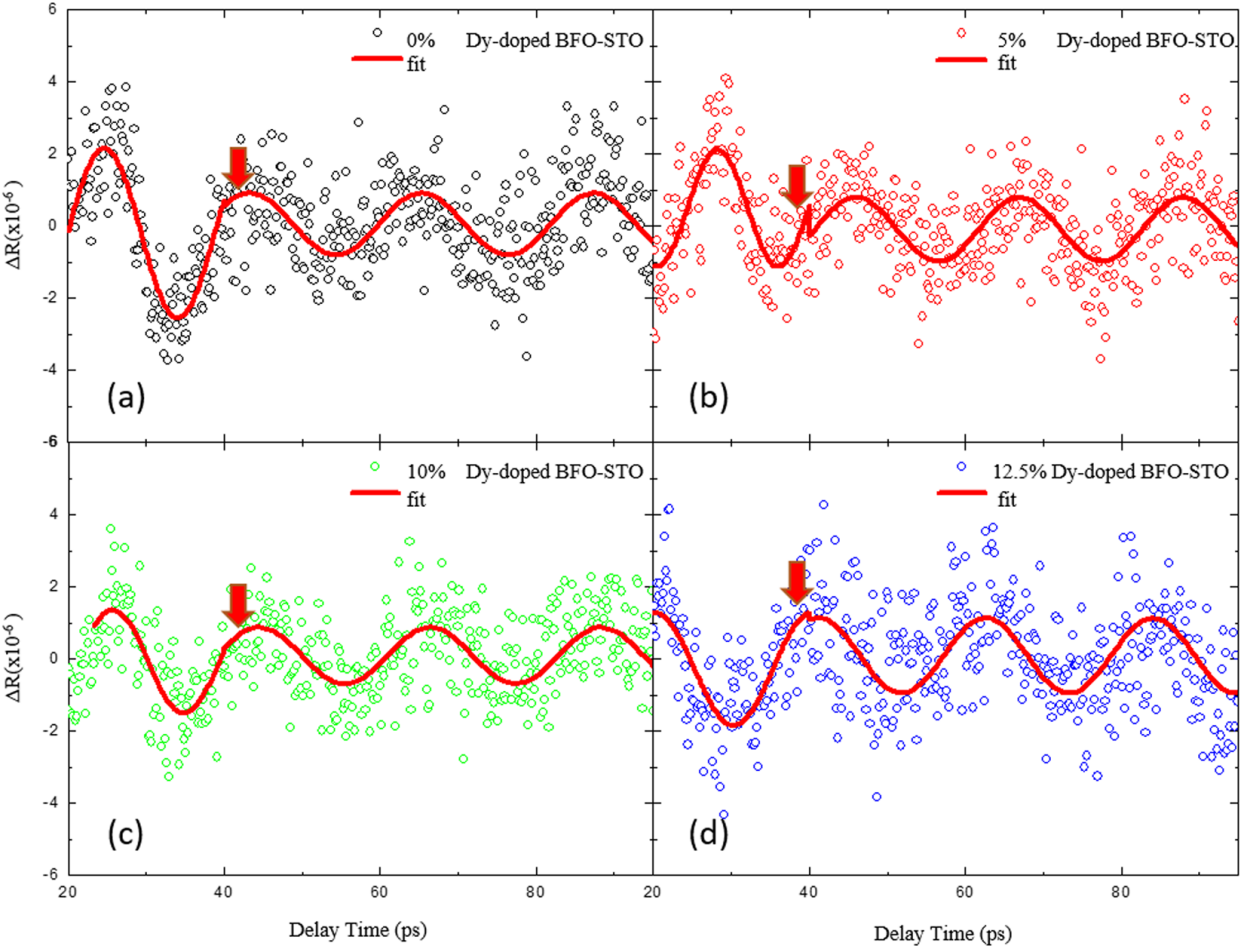
The extracted oscillations of BDFO films from their ultrafast transient reflectivity spectroscopy (Fig. 2(c)), with Dy-doping ratio of 0% (**a**), 5% (**b**), 10% (**c**), and 12.5% (**d**).

**Figure 4:**
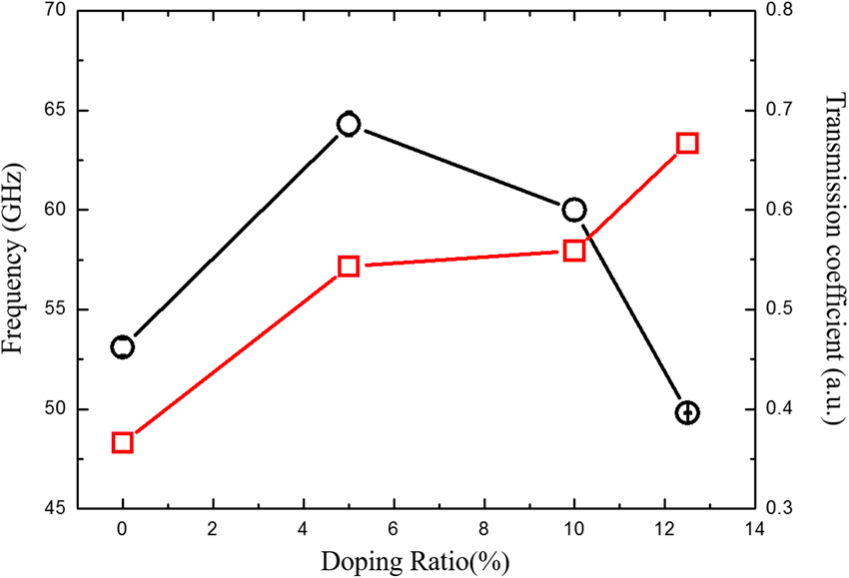
The fitted acoustic phonon frequency of DBFO films and transmission coefficient between DBFO films and STO substrate with various Dy-doping ratio.

In addition to the ultrafast dynamics of the structural phase transition in BDFO films, the ultrafast elastic response modulated by the substitution was observed. After subtracting the background decay, the oscillation feature of ΔR/R is obtained, which is shown in Fig. 3, the ultrafast strain pulse is generated by the relaxation of the optical phonon in about 15 ps, and then arrives in the substrate in the form of strain pulse^17,26^. As shown in Fig. 3(a), a discontinuity is observed in our fitting data, the amplitude and the frequency of the first oscillation are both larger than those of the following oscillations after about 30 picosecond. The first oscillation comes from the contribution from the coherent acoustic phonon in the BDFO film, and the following oscillation is contributed by the strain pulse propagation in SrTiO_3_ substrate, as a result, the following oscillation behaves a different amplitude and frequency as the initial one^27^. As shown in Fig. 4, it is seen that the acoustic phonon frequency is strongly related to the Dy-substitution ratio: the phonon frequency increases with the Dy-substitution ratio, when the substitution ratio is higher than 5%, the phonon frequency shows a decrease with the Dy-substitution ratio. This frequency dependent feature could be attributed to the structural phase transition caused by the Dy-doping, and the Hooke tensor is modulated correspondingly by the transition^19^. It should be noted that the acoustic phonon frequencies in Dy-doping BFO films are ranged from 50 GHz to 70 GHz, which is much higher than that in previous reports^17^. This discrepancy comes from the thinner BFO film used in present study, as the thickness of BFO film has close contact with the strain intensity introduced by the substrate. Here it is found that our 100-nm-thick BFO films has much larger acoustic phonon frequency than that in bulk-like BFO samples, which indicates that the strain intensity introduced by the substrate could modify the acoustic phonon frequency in BFO films greatly.

The transmission coefficient acoustic phonon can be calculated by

4 $${A}_{t}/{A}_{i}=\frac{2{\rho }_{s}{c}_{s}}{{\rho }_{s}{c}_{s}+{\rho }_{f}{c}_{f}},$$

which gives the energy transfer efficiency of the strain pulse at the interface of BDFO/STO. The A_i_ and A_t_ correspond the amplitude of the incident and transmitted acoustic waves, respectively, which are obtained here by the fitted function, the products of the density and sound velocity of the substrate and the film, ρ_s_c_s_ and ρ_f_c_f_ are the sound pressures in the substrate and BDFO film, respectively. As shown in Fig. 4, with increasing the Dy-doping ratio, the acoustic phonon transmission coefficient increases up to unity, which indicates the sound pressure in the BDFO film is well matched with that in the substrate at high Dy-doping ratio. The density and sound pressure in the film and substrate could be regarded as invariant, and the sound velocity is proportional to the Young’s modulus. By increasing the rare-earth doping ratio, the softening behavior of the Young’s modulus in BDFO occurs, which could result in an enhancement of local piezoresponse^26,28^.

## Conclusion

The ultrafast two-color pump probe technique is employed to investigate the Dy-doped BDFO films on STO substrate. It’s demonstrated that the Dy-doping could modulate the crystal structure of the BFO film and thus induce a structural and magnetic transition, which is uncovered by the ultrafast electron-phonon coupling and spin-lattice coupling. Meanwhile, the Dy-doping ratio dependent elastic tunability in the strained BFO is also observed, the ultrafast photo-induced coherent acoustic phonon transfer at the interface of BFO/STO demonstrates that the soften behavior of Young’s modulus in BDFO thin film is caused by the Dy doping. These findings thus provide direct evidence of the coupling between electron, spin and lattice degrees of freedom around the morphotropic phase boundary of Dy-doped BFO films.

## Methods

Bi_1−x_Dy_x_FeO_3_ (BDFO) (x = 0-0.125) epitaxial films were grown on single crystal SrTiO_3_ (STO) substrates by pulsed laser ablation of stoichiometric ceramic targets. The orientation of the STO substrates were 4° off the (001) plane along the <110> direction, to ensure a ferroelectric single-domain state in the BDFO films^29^.During growth, the substrate temperature is held at 650 °C under an oxygen partial pressure of 50 mTorr. The laser fluence was 1–1.5 J/cm^2^ with a repetition rate of 10 Hz, resulting in a growth rate of ~2 nm/min. All films have a thickness about 120 nm.

A reflection type pump probe set up is employed here which was described detailed in our previous publications^18^. The light source was provided by a commercial mode-locked Ti:sapphire laser (Spitfire Pro, Spectra-Physics) running at the repetition rate of 1 kHz, the pulse width of 120 fs, and the center wavelength of 800 nm. The pump beam with photo energy of 3.1 eV is obtained from a frequency-doubled fundamental beam (800 nm) in a 1-mm β-BaB_2_O_4_ crystal. The average pump fluence is about 6 mJ/cm^2^ which is 30 times larger than that of the probe. Both of the pump and probe beams were focused on the surface of the sample with a spot diameter about 200 μm^30^.
